# Irinotecan eluting beads-transarterial chemoembolization using Callispheres® microspheres is an effective and safe approach in treating unresectable colorectal cancer liver metastases

**DOI:** 10.1007/s11845-021-02629-9

**Published:** 2021-07-15

**Authors:** Guangsheng Zhao, Song Liu, Yuewei Zhang, Tong Zhao, Ruoyu Wang, Jie Bian, Jianlin Wu, Jun Zhou

**Affiliations:** 1 Cancer Interventional Center, Affiliated Zhongshan Hospital of Dalian University, Dalian, 116001 Liaoning China; 2 Cancer Interventional Center, Linyi Cancer Hospital, Linyi, 276001 Shandong China; 3 Hepatobiliary and Pancreatic Center, Beijing Tsinghua Changgung Hospital, Beijing, 102218 China; 4 Department of Radiology, The Second Affiliated Hospital of Dalian Medical University, Dalian, 116027 Liaoning China; 5 Department of Radiology, Affiliated Zhongshan Hospital of Dalian University, Dalian, 116001 Liaoning China

**Keywords:** Callispheres® microspheres, Colorectal cancer liver metastases, Efficacy, Irinotecan-eluting beads-transarterial chemoembolization, Safety

## Abstract

**Background:**

Callispheres® microspheres (CSM) are the first drug-eluting bead (DEB) product developed in China; meanwhile, DEB-transarterial chemoembolization (TACE) with CSM is effective and safe in the treatment of hepatocellular carcinoma and intrahepatic cholangiocarcinoma. However, the data regarding the role of irinotecan-eluting beads-TACE (DEBIRI-TACE) using CSM for colorectal cancer liver metastases (CRLM) treatment is limited. Therefore, the present study aimed to investigate the efficacy and safety of DEBIRI-TACE using CSM in the patients with unresectable CRLM.

**Methods:**

Totally, 42 unresectable CRLM patients treated with DEBIRI-TACE using CSM were continuously enrolled in this study. Postoperative treatment response (including complete response rate (CR), objective response rate (ORR), and disease control rate (DCR)), survival data (overall survival (OS)), liver function, and adverse events were documented during the follow-up.

**Results:**

CR, ORR, and DCR were 19.0%, 92.9%, and 100.0%, respectively, at month (M) 1; were 23.8%, 92.9%, and 97.6%, respectively, at M3; then were 14.3%, 78.6%, and 90.5%, respectively at M6. Regarding survival profiles, 1-year OS was 81.0%; 2-year OS was 58.5%; median OS was 25.0 months (95%CI: 19.3–30.7 months). Additionally, ALT and AST experienced an obviously increased trend at 4 days, but a declined trend at 7 days, while ALB and TBIL had no obvious change. No grade 3 or grade 4 adverse event was observed, and main adverse events included fever (95.3%), pain (57.1%), fatigue (50.0%), and nausea/vomiting (42.8%).

**Conclusion:**

DEBIRI-TACE with CSM achieves high treatment response, acceptable survival benefits, and good toleration in unresectable CRLM treatment.

## Introduction

Colorectal cancer ranks as the third most prevalent cancer with approximately 400,000 new cases worldwide annually [1]. It is estimated that about 50% patients diagnosed with colorectal cancer develop metastatic diseases, among which liver metastasis is regarded as one of the most common distant metastases [1, 2]. Mechanically, the metastatic process is a multistep event in which cancer cells escape from the primary tumor, survival in the circulation, then seed at distant sites, and grow, which is promoted by communications between tumor cells and immune cells via the secretion of cytokines, growth factors, and proteases remodeling the tumor microenvironment [3]. Currently, surgical resection of liver metastases is the only potentially curative therapy for colorectal cancer liver metastases (CRLM) patients, which contributes to a long-term survival benefit; however, only estimated 20% CRLM patients are eligible for resection [4]. For majority of unresectable CRLM patients, the management remains a clinical challenge; therefore, it is of great need to explore effective and safe treatment approach for CRLM treatment.

Transarterial chemoembolization (TACE) is a technique of therapeutic approach that includes drug delivery and embolization of agents into tumor-feeding arteries in order to prolong chemotherapy duration in targeted tumor and induce tumor ischemic necrosis, which is considered as an effective and alternative treatment for unresectable liver cancer [5, 6]. Drug-eluting bead TACE (DEB-TACE), a novel drug-delivering device in comparison with conventional TACE (cTACE), combines drugs and embolic materials in drug-loaded microspheres with the purposes of sustained chemotherapy release and increased local concentration of anti-tumor drugs [7, 8]. Existing evidence has demonstrated the application of DEB-TACE in the treatment of liver cancers, including CRLM, hepatocellular carcinoma (HCC), and intrahepatic cholangiocarcinoma (ICC) [9]. For example, one study indicates that irinotecan-loaded DEB-TACE presents increased survival benefit and less time for duration of improved life quality compared with standard chemotherapy in CRLM patients [6] In addition, Callispheres® microspheres (CSM) is the first polyvinyl alcohol (PVA) microsphere product developed in China, which can load several kinds of chemotherapeutic drugs (including irinotecan), and exhibits with several impressive properties (such as high drug-loading efficiency, stable drug-releasing profiles, multiple size) [10]. Previous researches have suggested that DEB-TACE with CSM is an effective and safe treatment approach in patients with unresectable HCC and ICC [11–14]. To date, limited data are available regarding the use of irinotecan-eluting beads-transarterial chemoembolization (DEBIRI-TACE) using CSM for the treatment of CRLM.

Therefore, in the current study, we evaluated the efficacy and safety of DEBIRI-TACE using CSM in patients with unresectable CRLM.

## Methods

### Study population

This study was carried out with the approval by Institutional Review Board. From January 2017 to March 2018, 42 unresectable CRLM patients treated with DEBIRI-TACE were continuously enrolled in this study. The inclusion criteria were as follows: (1) liver metastasis after colorectal cancer surgery, which was confirmed by liver puncture biopsy pathology; (2) unresectable metastatic lesion in liver, or patients refused to undergo surgery; (3) single or multiple measurable lesions in the liver, suggested by imaging examination; (4) age more than 18 years; (5) scheduled for undergoing DEBIRI-TACE; (6) performance score ≤ 2; and (7) expected survival time more than 6 months. The exclusion criteria included (1) contraindications for the interventional treatment; (2) allergic to embolism materials or drugs used in the study; (3) renal, cardiac, or pulmonary dysfunction before the operation; (4) unable to be followed up regularly; and (5) female patients in pregnant or lactating. All enrolled patients provided the written informed consents.

### Data collection

Demographic and clinical characteristics of patients were documented after examinations, which mainly included age, gender, Karnofsky performance status (KPS) score, Child-Pugh stage, number of intrahepatic metastases, tumor differentiation, time after resection of primary lesion, and history of systemic chemotherapy.

### DEBIRI-TACE technology

Drug-loading procedures were performed before TACE operation. CSM (Jiangsu Hengrui Pharmaceutical Co. Ltd, Lianyungang, Jiangsu, China), with a diameter of 100–300 μm, were used to load irinotecan (Jiangsu Hengrui Pharmaceutical Co. Ltd, Lianyungang, Jiangsu, China), as follows: the CSM were mixed with irinotecan (200 mg) at room temperature, shaken once every 5 min for 30 min, then mixed with the non-ionic contrast agent iodixanol injection at a ratio of 1:1. After completion of drug-loading procedures, TACE operation was followed. The right femoral artery was successfully punctured using the Seldinger technique, then a right-heart catheter was routinely introduced into the artery, aimed for undergoing the angiography of the celiac trunk and common hepatic artery. Under digital subtraction angiography fluoroscopy, the prepared drug-loaded CSM solution was slowly injected into the artery supplying blood to the tumor (1 mL was administered each time using a syringe and injected at a speed of 1 mL/min). If the tumor diameter was > 5 cm or a bottle of drug-loaded CSM was still insufficient for complete embolism, the gelatin sponge particles (150~350 μm, Hangzhou Ailikang Pharmaceutical Technology Co., Ltd, Hangzhou, Zhejiang, China) were used for supplementary embolism. The endpoint of chemoembolization was either (1) disappearance of tumor staining, (2) stagnancy of contrast agent in the tumor target vessel, or (3) stagnancy of contrast agent after three cardiac cycles.

### Efficacy evaluation and adverse event observation

We performed enhanced computed tomography (CT)/magnetic resonance imaging (MRI) at 1 month after the operation to observe the lesion size, degree of necrosis, and presence of new lesions, which was then performed every 2 to 3 months. The Modified Response Evaluation Criteria in Solid Tumors (mRECIST) criteria were applied to evaluate the postoperative tumor response in the liver at month 1 (M1), month 3 (M3), and month 6 (M6), with a focus on observing the scope of tumor activity, comprehensively evaluating the outcome of intervention, and determining whether the interventional treatment should be delivered again. In addition, the liver function before and after interventional treatment (4 days and 7 days post-operation) was evaluated. Adverse events that occurred during the study were recorded and graded according to the National Cancer Institute Common Terminology Criteria for Adverse Events Version 3.0.

### Survival assessment

Continuous follow-up observation was conducted for all patients. The follow-up was stopped until the patients’ death or the end of the study. The survival rate and survival time of patients after the interventional procedure were observed and summarized. Overall survival (OS) was defined as duration from the DEBIRI-TACE treatment to the patients’ death.

### Statistical analysis

SPSS statistical software 20.0 (IBM, Chicago, Illinois, USA) was used for statistical analysis of the detected data. Descriptive statistical analyses were performed for the clinical data. The Kaplan–Meier method was used to plot the survival curve, and the median OS was calculated.

## Results

### Demographic and clinical characteristics

In these included CRLM patients, the mean age was 56.5 ± 8.2 years (Table 1). The number of patients ≥ 60 years and < 60 years were 13 (31.0%) and 29 (69.0%), respectively. Furthermore, the percentage of male and female included were 64.3% and 35.7%, respectively. Moreover, there were 37 (88.1%) and 5 (11.9%) patients with Child-Pugh stage A and B, respectively. Meanwhile, there were 12 (28.6%) patients with single intrahepatic metastases and 30 (71.4%) patients with multiple intrahepatic metastases, respectively. More detailed information of CRLM patients is shown in Table 1.

**Table 1:** Demographic and clinical characteristics of CRLM patients

Items	CRLM patients (*N* = 42)
Age (years), mean ± SD	56.5 ± 8.2
≥ 60 years, No. (%)	13 (31.0)
< 60 years, No. (%)	29 (69.0)
Gender, No. (%)	
Male	27 (64.3)
Female	15 (35.7)
KPS score, No. (%)	
≤ 80	10 (23.8)
> 80	32 (76.2)
Child-Pugh stage, No. (%)	
A	37 (88.1)
B	5 (11.9)
Number of intrahepatic metastases, No. (%)	
Single	12 (28.6)
Multiple	30 (71.4)
Tumor differentiation, No. (%)	
Well	24 (57.2)
Moderate	14 (33.3)
Poor	4 (9.5)
Time after resection of primary lesion, No. (%)	
< 2 years	28 (66.7)
≥ 2 years	14 (33.3)
Previous systemic treatment, No. (%)	
Yes	30 (71.4)
No	12 (28.6)
Number of interventions, mean ± SD	2.78 ± 1.18

*CRLM* colorectal cancer liver metastasis, *SD* standard deviation,* KPS* Karnofsky Performance Status

### Treatment response

**Fig. 1:**
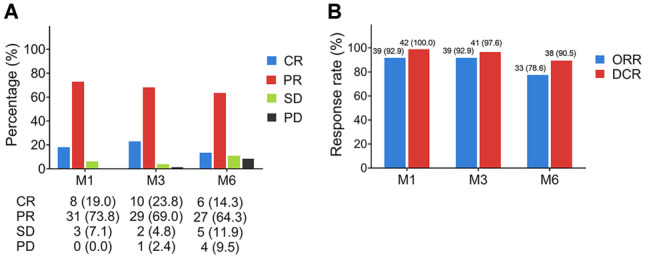
**b** CR, PR, SD, and PD in CRLM patients treated with DEBIRI-TACE. **A** ORR and DCR in CRLM patients treated with DEBIRI-TACE. **B** CR, complete response; PR, partial response; SD, stable disease; PD, progressive disease; ORR, objective response rate; DCR, disease control rate; CRLM, colorectal cancer liver metastases; DEBIRI-TACE, irinotecan eluting beads-transarterial chemoembolization

**Fig. 2:**
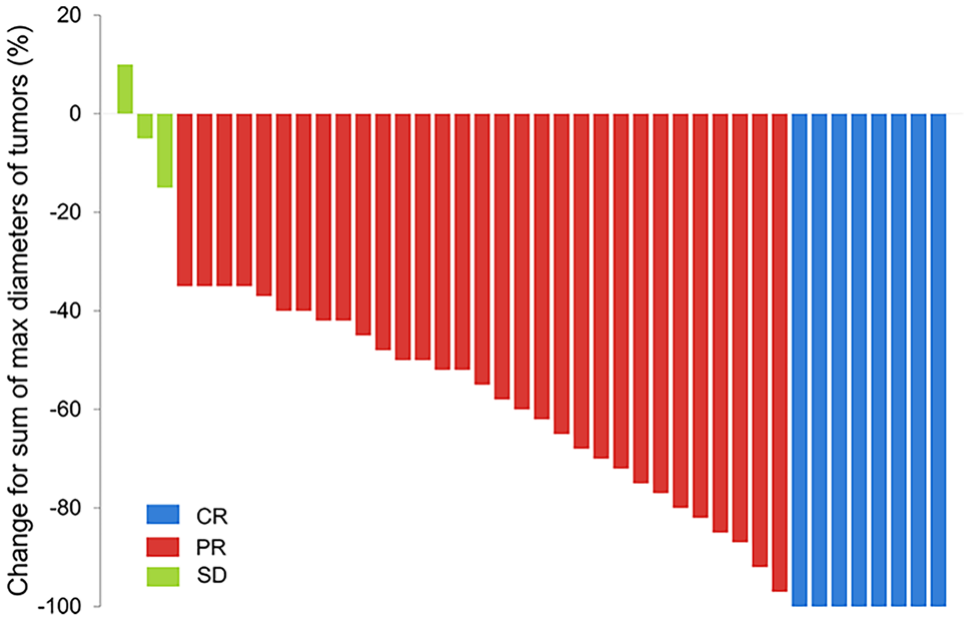
Change of tumor diameters at M1. CRLM, colorectal cancer liver metastases; DEBIRI-TACE, irinotecan-eluting beads-transarterial chemoembolization; CR, complete response; PR, partial response; SD, stable disease; M1, month 1

Complete response (CR), partial response (PR), stable disease (SD), and progressive disease (PD) were 19.0%, 73.8%, 7.1%, and 0.0%, respectively, at M1; were 23.8%, 69.0%, 4.8%, and 2.4%, respectively, at M3; and were 14.3%, 64.3%, 11.9%, and 9.5%, respectively, at M6 (Fig. 1a). In addition, objective response rate (ORR) and disease control rate (DCR) were 92.9% and 100.0%, respectively, at M1; were 92.9% and 97.6%, respectively, at M3; and were 78.6% and 90.5%, respectively, at M6 (Fig. 1b). In detail, the change for sum of max diameter of tumor within M1 in each patient is shown in Fig. 2.

### Survival profiles

**Fig. 3:**
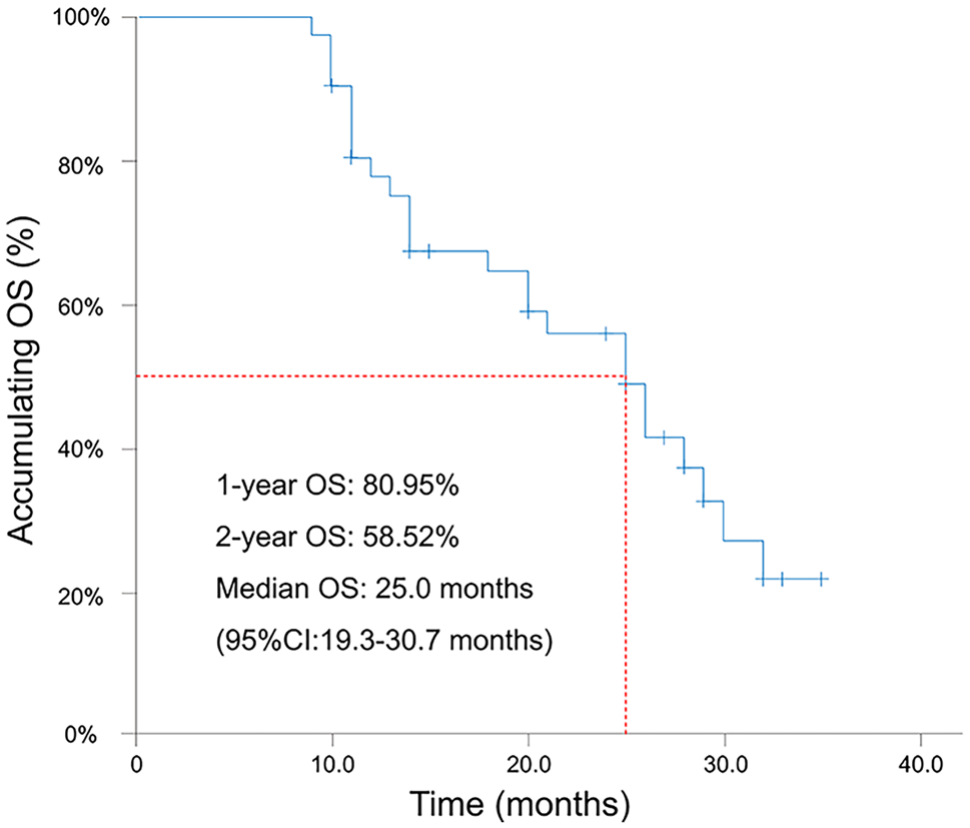
Accumulating OS. OS, overall survival; CRLM, colorectal cancer liver metastases; DEBIRI-TACE, irinotecan-eluting beads-transarterial chemoembolization; CI, confidence interval

Continuous follow-up observation was conducted in all patients, which observed that 1-year OS was 80.95%; 2-year OS was 58.52%; median OS was 25.0 months (95%CI: 19.3–30.7 months) (Fig. 3).

### Liver function indexes

**Fig. 4:**
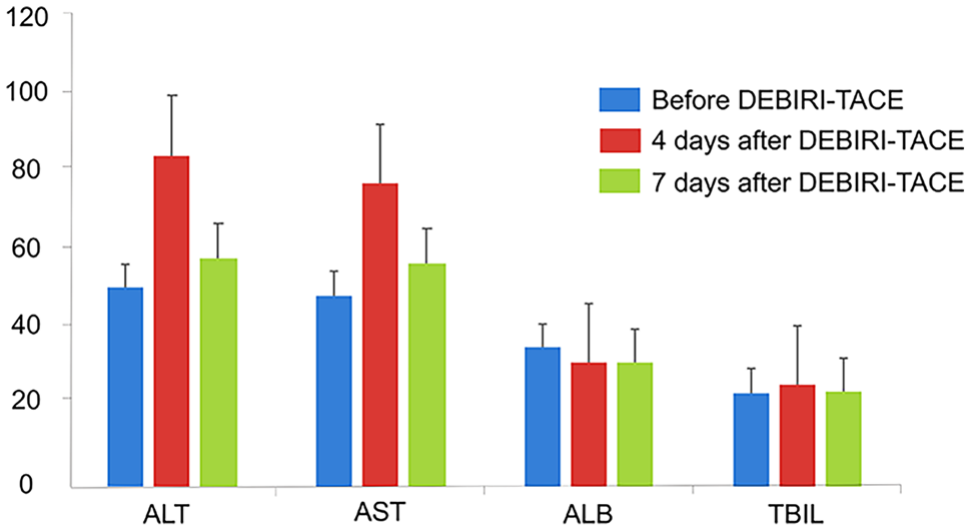
ALT, AST, ALB, TBIL at 4 and 7 days. CRLM, colorectal cancer liver metastases; DEBIRI-TACE, irinotecan-eluting beads-transarterial chemoembolization; ALT, alanine aminotransferase; AST, aspartate aminotransferase; ALB, albumin; TBIL, total bilirubin

Numerically, alanine aminotransferase (ALT) and aspartate aminotransferase (AST) underwent a great increased trend at 4 days after DEBIRI-TACE, but experienced a declined trend at 7 days after DEBIRI-TACE (Fig. 4). However, albumin (ALB) and total bilirubin (TBIL) did not experience obvious changes at 4 days and 7 days after DEBIRI-TACE.

### Adverse events

There was no grade 3 or grade 4 adverse events that occurred during the follow-up, and all adverse events were mild and well-tolerable (Table 2). In detail, the total incidences of fever, pain, fatigue, nausea/vomiting, liver function damage, bone marrow suppression, delayed-onset diarrhea, and biloma were 95.3%, 57.1%, 50.0%, 42.8%, 28.6%, 11.9%, 2.4%, and 2.4%, respectively. More information about adverse events is shown in Table 2.

**Table 2:** Adverse events

Items	Total	Grade 1	Grade 2	Grade 3	Grade 4
Fever, No. (%)	40 (95.3)	27 (64.3)	13 (31.0)	0 (0.0)	0 (0.0)
Pain, No. (%)	24 (57.1)	20 (47.6)	4 (9.5)	0 (0.0)	0 (0.0)
Fatigue, No. (%)	21 (50.0)	20 (47.6)	1 (2.4)	0 (0.0)	0 (0.0)
Nausea/vomiting, No. (%)	18 (42.8)	15 (35.7)	3 (7.1)	0 (0.0)	0 (0.0)
Liver function damage, No. (%)	12 (28.6)	11 (26.2)	1 (2.4)	0 (0.0)	0 (0.0)
Bone marrow suppression, No. (%)	5 (11.9)	5 (11.9)	0 (0.0)	0 (0.0)	0 (0.0)
Delayed-onset diarrhea, No. (%)	1 (2.4)	1 (2.4)	0 (0.0)	0 (0.0)	0 (0.0)
Biloma, No. (%)	1 (2.4)	1 (2.4)	0 (0.0)	0 (0.0)	0 (0.0)

### A typical case

**Fig. 5:**
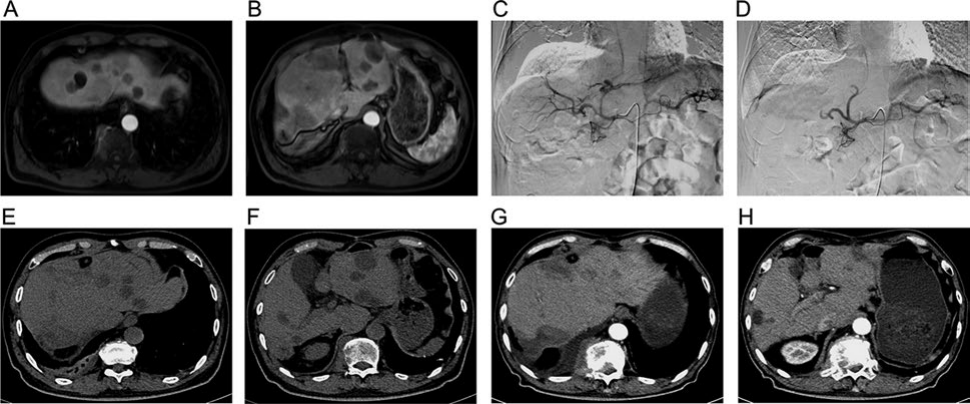
A typical case treated with DEBIRI-TACE. The intrahepatic metastatic lesions by enhanced MRI before DEBIRI-TACE treatment **A**, **B**. The detection by arteriography during the DEBIRI-TACE treatment **C**. The detection of secondary arterial branch and tumor staining by arteriography after the DEBIRI-TACE treatment **D**. The regression of tumor lesion by scan CT after the DEBIRI-TACE treatment **E**, **F**. Tumor detection by enhanced CT after 6 months post DEBIRI-TACE treatment **G**,** H**. DEBIRI-TACE, irinotecan-eluting beads-transarterial chemoembolization; MRI, magnetic resonance imaging; CT, computed tomography

A patient was diagnosed to be liver metastasis characterized by diffuse distribution after colorectal cancer surgery, who had the treatment history including surgical resection, radiofrequency ablation, systematic chemotherapy, and microwave ablation. Before the treatment of DEBIRI-TACE, enhanced MRI observed multiple intrahepatic metastatic lesions (Fig. 5a, b). During the treatment of DEBIRI-TACE, arteriography showed the slight tumor staining in the arterial and liver parenchymal phase (Fig. 5c). After the embolization by DEBIRI-TACE, arteriography showed stagnancy of contrast agent in the secondary branches of the intrahepatic artery as well as the complete disappearance of the tumor staining (Fig. 5d). At 4-day post-treatment, CT found the obvious decreased intensity of intrahepatic tumor lesions (Fig. 5e, f). Furthermore, at 6-month post-treatment, the follow-up enhanced CT showed that tumor completely disappeared, and there was no enhancement of residual lesions (Fig. 5g, h).

## Discussion

With the development of biotechnology and novel material investigation, several DEBs, such as DC beads, LC beads, HepaSphere microspheres, have been introduced and applied for DEBIRI-TACE treatment [15]. Existing evidence have indicated the application of DEBIRI-TACE with these beads in the treatment of CRLM with ORR ranging from 70 to 80% at 1 month [14–17]. For example, after treatment of TACE using irinotecan-loaded super-absorbent polymer microspheres, CR and PR were 24% and 48% at 3 months, and the mean follow-up after last TACE is 14.6 months with median of 8 months [17]. Furthermore, CSM is a novel microsphere product developed in China, while its application in CRLM treatment has not been conducted before. Therefore, we carried out the current study to fill the blank.

In terms of DEBIRI-TACE using CSM, CR, ORR, and DCR in our study were 19.0%, 92.9%, and 100.0%, which were numerically increased compared with the treatment response in previous studies which used other beads (CR: 15%; ORR: 45%; DCR: 25% in patients treated with DEBIRI-TACE using LC beads) [18]. This finding suggested the potentially feasible and superior role of CSM for the DEBIRI-TACE treatment. The possible reason might be as follows: (1) the difference between our study and those previous studies (including sample size, patients’ characteristics, and etc.) might lead to the discrepancy of treatment response. (2) Considering the outstanding features of CSM in drug loading and release [10, 11], DEBIRI-TACE using CSM enhanced directed intra-tumor chemotherapy concentration and kept more sustained drug release, thereby effectively contributing to the better treatment response in unresectable CRLM patients.

Furthermore, regarding survival profiles, in the current study, it was observed that 2-year OS rate was 58.52% and the median OS was 25.0 months in patients treated with DEBIRI-TACE using CSM, which was numerically longer compared with the OS (approximately 22.0 months) in CRLM patients who received DEBIRI-TACE using other beads (DC beads, LC beads, HepaSphere microspheres) [6, 14–17, 19]. The possible reasons might include the difference between our study and the prior trials (such as sample size, clinical characteristics of patients, and etc.). In addition, given the positive association of treatment response with survival profiles [20], CRLM patients exhibited longer survival which might be due to favorable treatment response by DEBIRI-TACE using CSM.

Notably, our study provided evidence regarding the limited hepatic toxicity of DEBIRI-TACE in CRLM patients, which might be due to the history of concomitant cirrhosis, chemotherapeutic drugs, and the process of ischemia–reperfusion in the liver [21, 22]. Additionally, the adverse events that occurred were all in grade 1 or 2, suggesting that DEBIRI-TACE using CSM presented a mild and well-tolerable safety profile. In detail, adverse events included fever, pain, fatigue, nausea/vomiting, liver function damage, bone marrow suppression, delayed-onset diarrhea, and biloma. According to previous evidence, myelosuppression, diarrhea, and biloma were common caused by active metabolite of irinotecan in the bowel, which was related to a cholinergic surge from the inhibition of acetylcholinesterase, delayed diarrhea syndrome [22]. In addition, other adverse events (such as fever, pain, fatigue, nausea/vomiting) were frequent post-embolic syndrome and could be self-resolved. These findings were in line with the previous studies that DEB-TACE using CSM presented well-tolerable adverse events in treatment of other liver malignancies [11, 12].

However, the present study still existed several limitations. (1) Considering the limited sample size (42 patients enrolled) in the current study, more patients from multiple regions were essential for validating the results. (2) As the most patients were from neighboring regions, and they went back to local hospital for disease surveillance, therefore, the information about tumor recurrence was not documented precisely in majority of CRLM patients, which needed to be explored in further study. In addition, the follow-up duration in our study was relatively short, which needed further duration for investigation. (3) Some bias, such as the differences in the operation skills of surgeons, patients’ socioeconomic status, treatment history, were not included in the current study, which might influence the results in the current study. (4) Given that our study was a single-armed observational clinical research, further randomized controlled trials which compared CSM with other kinds of beads (DC beads, LC beads, HepaSphere microspheres) were required to recognize the optimal role for the CRLM management. (5) Further studies included cTACE as control therapy were needed to compare the efficacy of DEBIRI-TACE with those of cTACE in unresectable CRLM patients.

In conclusion, DEBIRI-TACE using CSM represents to be an effective and safe treatment approach in the management of unresectable CRLM, suggesting its role as an additional treatment option for unresectable CRLM.
